# Communication: The formation of rarefaction waves in semiconductors after ultrashort excitation probed by grazing incidence ultrafast time-resolved x-ray diffraction

**DOI:** 10.1063/1.4963011

**Published:** 2016-09-23

**Authors:** S. Höfer, T. Kämpfer, E. Förster, T. Stöhlker, I. Uschmann

**Affiliations:** 1 Institut für Optik und Quantenelektronik, Friedrich-Schiller-Universität Jena, 07743 Jena, Germany; 2 Helmholtz-Institut Jena, 07743 Jena, Germany; 3 GSI-Helmholtzzentrum für Schwerionenforschung GmbH, 64291 Darmstadt, Germany

## Abstract

We explore the InSb-semiconductor lattice dynamics after excitation of high density electron-hole plasma with an ultrashort and intense laser pulse. By using time resolved x-ray diffraction, a sub-mÅ and sub-ps resolution was achieved. Thus, a strain of 4% was measured in a 3 nm thin surface layer 2 ps after excitation. The lattice strain was observed for the first 5 ps as exponentially decaying, changing rapidly by time and by depth. The observed phenomena can only be understood assuming nonlinear time dependent laser absorption where the absorption depth decreases by a factor of twenty compared to linear absorption.

## I. INTRODUCTION

Today, the performance of semiconductor devices in modern electronics and optics reaches the physical limits regarding temporal dynamics and spatial structure. Fundamental physical properties such as charge carrier mobilities and deformation potential determine today the powerful capability of integrated circuits. Such properties are determined by the detailed treatment of the semiconductor lattice such as the lattice strain which can significantly increase the drive current and the carrier mobility used in modern planar transistor technology.^1–3^ The temporal evolution of electronic processes after electronic excitation can be studied by ultrafast optical quantum electronics where the time resolution can be orders of magnitude faster than for electronic diagnostics. Ultrafast carrier production excited by short laser pulses started with picoseconds duration forty years ago.^4,5^ Since this time the evolution of carriers could be controlled by laser pump-laser probe experiments, studying excitation of very dense electron-hole (e-h) plasmas, ambipolar carrier diffusion after the excitation, recombination processes like optical phonon and acoustical phonon production, as well as Auger decay. Anyway, the transient lattice parameters like strain can not be studied directly by optical methods. Alternatively such studies have been established in the last decade by ultrashort x-ray diffraction. Today, there are three sources providing x-ray pulses with sub-ps pulse duration at a wavelength around 1 Å. The free-electron laser^6,7^ and the slicing sources^8^ are large facilities, but with limited beam time for experiments. The only laboratory sized short pulse x-ray source at present is the laser plasma x-ray source,^9–12^ with a pulse duration of about 100 fs.^13,14^ The present material indium antimonide (InSb) was studied in several experiments by time resolved diffraction. Sound waves were recorded for longer time delays and lower dynamic range.^15–18^ However, no results were presented for the first picoseconds when the energy is transferred to the lattice. Representative theoretical contributions to this field are time dependent descriptions of relaxation of highly excited semiconductors.^19–22^ With the help of rate equation, the time dependent evolution of carriers, and the electron and lattice temperatures were simulated and compared to the experiments.^23^

## II. EXPERIMENTAL DETAILS AND RESULTS

In this work, the excitation of a semiconductor by an ultrashort laser pulse below the damage threshold is investigated. The energy of the band gap of InSb is 0.17 eV; this is well below the photon energy of 1.5 eV of the Ti:sapphire laser used for excitation. By exciting InSb with a flux of 10 mJ/cm^2^ up to 10^21^ e-h pairs are excited simultaneously in the sample within the laser pulse duration of 60 fs. The carriers are created in a thin surface layer. After excitation, the electrons have a high kinetic excess energy. Energy is transferred by electron-electron collisions during the first picoseconds after the excitation. With the decreasing kinetic energy of the electrons after ≤1 ps, electron-phonon collisions and diffusion become dominant and the lattice temperature starts to increase. Several picoseconds later, the temperature of the lattice and of the electrons are in equilibrium. Beside diffusion, also recombination of e-h pairs and Auger recombination decrease the number of free electrons. The higher temperature and the dense free electron plasma lead to stress in the crystal of several MPa.^19^ The consequence of the stress is a deformation of the crystal lattice that was measured by time-resolved x-ray diffraction.

To adapt the excited and the probed volume, either a thin layer can be probed^24^ or asymmetric reflections can be used.^15,16,25^ In the case of asymmetric reflection, the diffracting lattice planes are not parallel to the crystal surface and incidence and emergence angles are different. By choosing a proper combination of crystal surface, x-ray energy, and reflecting lattice planes, a gracing incidence setup is possible. We applied the 220 reflection of a InSb (111) surface, with Ti-K*α*-radiation having a photon energy of 4510 eV. The Bragg angle amounts to 36.9°, the angle of emergence is 2°, and the extinction depth is 60 nm, compared to 600 nm for the symmetric (111) reflection.

**FIG. 1.**
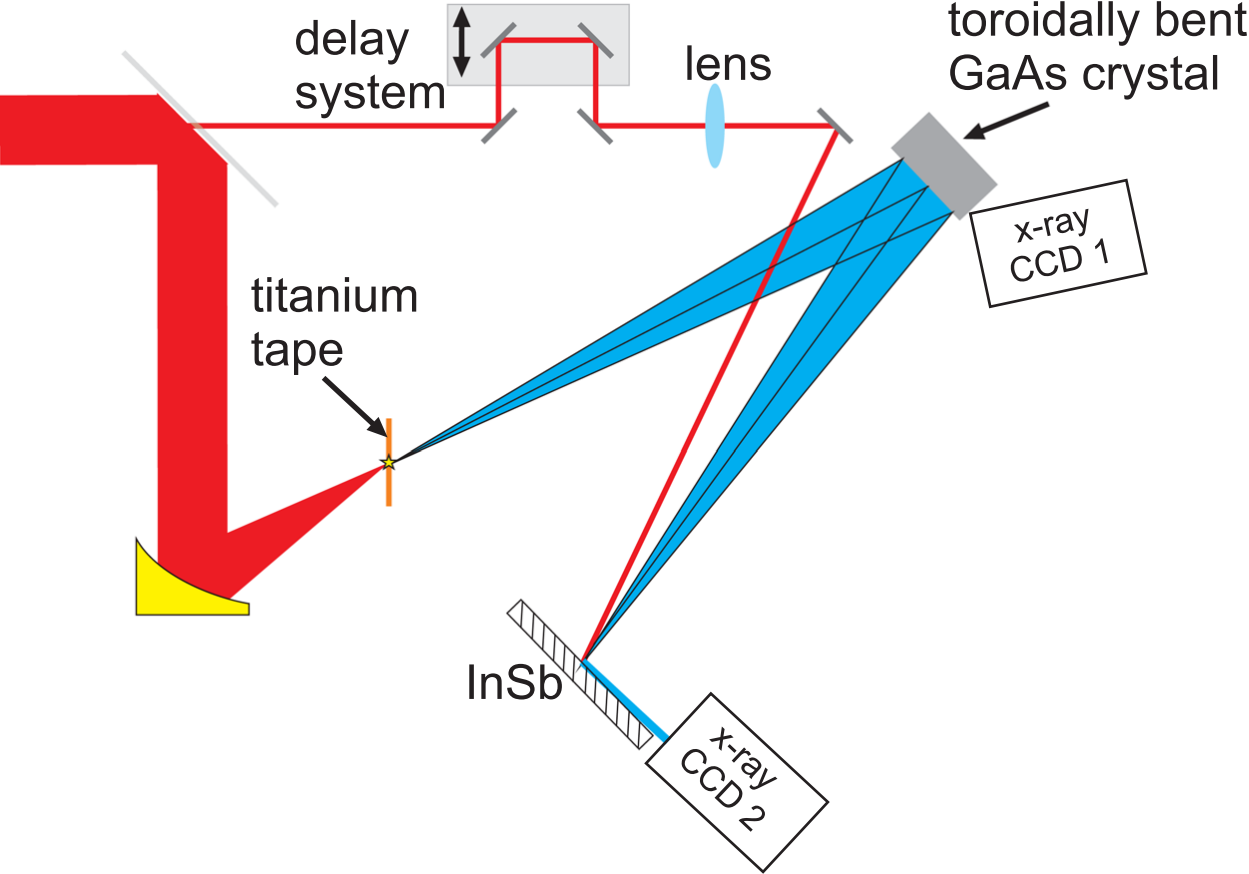
Pump-probe setup to measure transient structural changes of InSb (see text for details).

Figure 1 shows the setup for the optical pump x-ray probe experiment. By focusing Ti:sapphire laser pulses with 1 kHz repetition rate in vacuum on a titanium tape with an intensity of 8 × 10^16^ W/cm^2^, short x-ray pulses were generated. The isotropically emitted Ti-K*α* x-ray photons are focused on the sample by a toroidally bent GaAs crystal to a diameter of 110 *μ*m.^26^ The x-ray source and the crystal sample are placed on the Rowland circle of the toroidally bent GaAs crystal in order to probe the sample by a convergent monochromatic spherical wave. Two back-illuminated deep depletion ANDOR 420DX-BD-DD CCDs were used as detector.^27^ The first CCD was used to normalize the number of measured photons, and the second collects the diffracted photons from the sample. The acquisition time of the CCD's was chosen to ensure to work in the single photon counting mode^27^ in order to remove background photons of other energies. A pump beam having a well defined delay to the x-ray probe is focused to the same sample position as the probe. The pump beam is focused to a diameter of 500 *μ*m. At each temporal delay, one image with laser excitation and one without laser excitation were recorded. We found that the processes were 100% reversible. Several images were summed up for the same delay.

**FIG. 2.**
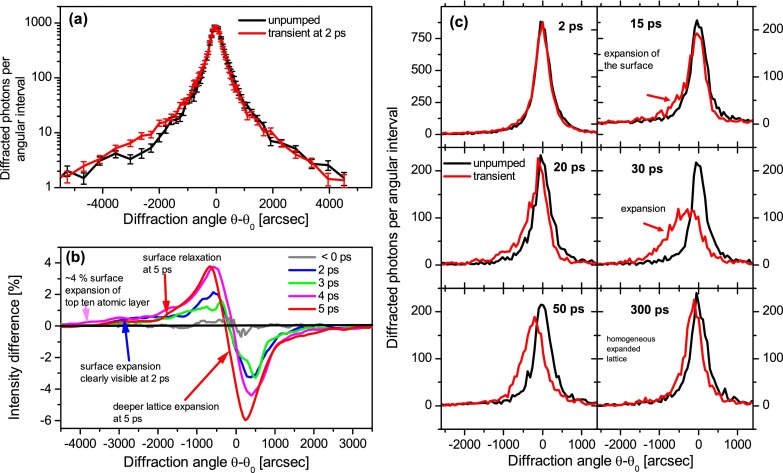
Time dependent rocking curves of the asymmetric InSb 220 reflection, probe depth: 60 nm, (a) rocking curves for the non perturbed and perturbed lattice, (b) intensity difference between perturbed and unperturbed curves for negative delays <0 ps, 2 ps, 3 ps, 4 ps, and 5 ps delay, (c) transient rocking curves for delays between 2 ps and 300 ps compared to the nonperturbed case, laser flux 10 mJ/cm^2^.

The integration along the diffraction peak yields the angular dependent diffracted intensity, the so called rocking curve. In Figure 2(a), two rocking curves are shown, first for the unperturbed crystal (black) and second for a probe delay 2 ps after excitation (red). At 2 ps, a small shift of the wings of the rocking curve and a small increase of the diffracted intensity at angles smaller than −2000″ become observable due to the logarithmic scaling and a dynamic range of three orders of magnitude. For better visualization of the small changes, a moving average filter was applied. The difference between the transient and the unperturbed curve is presented in Figure 2(b) for delay times up to 5 ps. The negative delays show a difference smaller than 0.5%, which means the crystal is fully recovered after 1 ms. In contrast, all other delays show a similar modification, a positive difference for smaller diffraction angles and negative difference for positive angles. Both differences grow by the increasing delay time from the excitation to 5 ps. The modification represents a shift of the rocking curves as well as an increase of the FWHM. Furthermore, a long tail larger than 3000″ at smaller angles occurs at times shorter than 4 ps. In the low angle wing of the rocking curve 400 photons are more diffracted in the perturbed than in the unperturbed crystal, with the total number of 9900 photons and the extinction depth of 60 nm, a strained surface layer of only 10 atomic layers can be estimated, with a maximum strain of ≥3%. This strain is more than one order of magnitude larger than the strain reported in previous measurements and was never observed for bulk crystals at such early times after a non-destructive excitation of the target. The minimal measurable peak shift is 20″ at 5 ps delay. 30 ps after excitation, the probed volume has a strong expanded lattice. The maximum change in the peak position occurs at 30 ps after excitation with a peak shift of 250″ which corresponds to a strain dominated by the heated lattice of 9.1 × 10^−4^. This corresponds to a sample with a temperature of 90 °C. At longer delay times, the lattice starts to relax and the rocking curve shifts back to the position of the original curve. The width gets narrower as well as the peak reflectivity is strongly increasing regarding to a reduction of the remaining strain. By using another 311 reflection with a much larger probing depth, we found that the lattice starts to be compressed 50 ps after excitation at a depth of about 200 nm.

## III. DISCUSSION

**FIG. 3.**
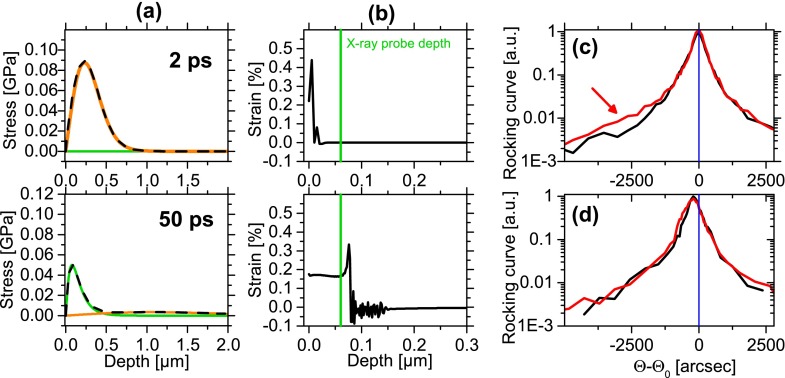
Simulated dynamic at the selected times 2 ps (top row) and 50 ps (bottom row). (a) Stress with the contributions by the electrons (orange) and the heated lattice (green) and (b) the resulting strain profiles. (c) and (d) Comparison of the simulated transient rocking curves (black) at 2 ps and 50 ps with the measurements (red) also presented in Figure 2. Please note in (c) that the strain induced intensity in the low angle wing (red arrow) is not reproduced by the simulation.

In order to compare our time-resolved x-ray diffraction data with theoretical results, the physical system was simulated in three stages. First, the dynamics of the semiconductor after excitation with an ultrashort laser pulse was treated by an approach by Lietoila and Gibbons.^20,21^ For a semiconducting sample, the following excitation and relaxation processes are important: Initially, there is charge carrier excitation by single and multiple photon absorption. The thermalization of the carrier distribution by carrier-carrier scattering has to be treated. Further carrier absorption by radiative and non-radiative recombination as well as carrier diffusion with the associated energy transport occurs. Then, energy is transferred from carriers to phonons by carrier-phonon scattering. Second, up to 10 ps, the stress is induced almost exclusively by the electronic contribution whereas for later times the lattice temperature dominated the stress (see Figure 3(a)). The stress results in a lattice deformation, i.e., compression and expansion, the deviation of the deformation is the strain *η* (see Figure 3(b)). The temporal and spatial evolution of the strain is described by the wave equation. Both steps are solved by time and space dependent differential equations. Due to the experimental conditions, the physical problems are treated one-dimensional in space.^23^ Third, the diffraction signal was calculated using the dynamical diffraction theory of the deformed crystals.^28^ In order to compare theory and experiment, the calculated rocking curves are convoluted with the apparatus function determined from nonperturbed rocking curves and deconvoluted by the InSb 220 reflection curve. The observed rocking curves at late delay times of ≥5 ps can be well reproduced by the simulation (see Figure 3(d)). With the model described above, we cannot reproduce the measured changes, in particular, the high strain on the surface for delays ≤5 ps (see Figure 3(c)).

**FIG. 4.**
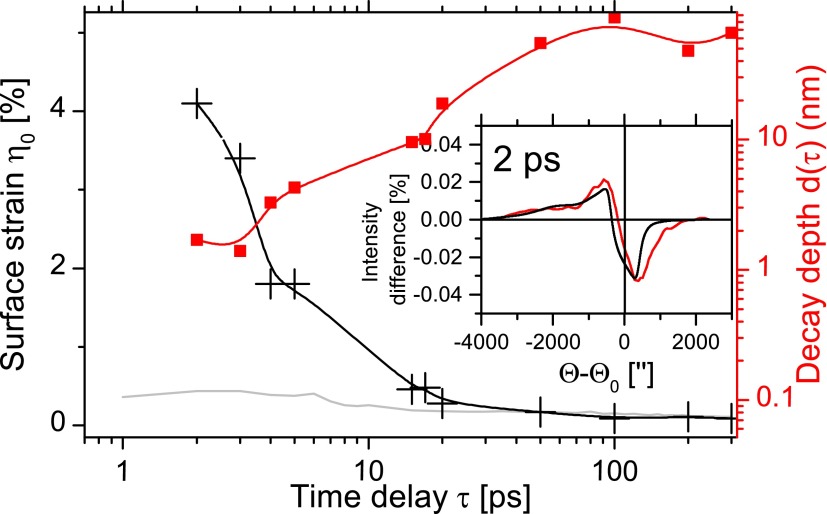
Transient progression of the surface strain *η*_0_(*τ*) (black) and the decay depth d(*τ*) (red), see Eq. (1). The maximal strain calculated in the simulation is shown for comparison (gray) (see Figure 3(b)). Whereas the simulation of the strain agrees well for *τ* > 20 ps, there is a strong difference at early times (2 ps…20 ps). The inset shows the reproduction of the 2 ps rocking curve profiles defined as the difference of intensity as shown in Figure 2(b) (red: measurement; black: adapted exponential strain).

To get a quantitative profile of the strain from the measured rocking curves, an exponential decay for the strain η(z,τ) was assumed, see Equation (1). The surface strain $\eta_{0}(\tau )$ and the decay depth d(τ) were adapted to the measured rocking curves at the different time delays, and the adapted values are presented in Figure 4,

$$\eta (z,\tau )=\eta_{0}(\tau )\cdot \text{exp}(-z/d(\tau )).$$ (1)

The rocking curves assuming an exponentially decaying strain reproduces the experimental data very well; exemplary, the curves at 2 ps are shown in the inset of Figure 4. At a delay time of 2 ps, the deformation has a decay depth of 1.7 nm and a surface strain of 4%, decreasing within 20 ps by a factor of 20. The values at 2 ps are in good agreement with the estimated values from Figure 2.

In order to explain the strong lattice deformation at the surface for early times, we calculated the time and space dependent absorption of the ultrashort intense laser pulse. By assuming the usual linear absorption, the absorption depth for the laser photons in InSb is 100 nm and up to 10^21^ e-h pairs per cm^3^ excited and 35% of the energy is reflected. This calculation neglects the change of reflectivity and absorption by instantaneously generated e-h pairs and is only valid at low intensities.^22^ For correct calculations, including the transient change of reflectivity and absorption due the e-h pairs the reflectivity and absorption was calculated time- and depth dependently. The relation between the electron density and the dielectric function is described by the Drude model.^29^ From the dielectric function, the reflectivity is calculated by the Fresnel equations and the absorption by the imaginary part of the refractive index. With this, the electron density dependent reflectivity at the surface and the absorption at each point in depth are calculated from the electron density of the previous time step. Each absorbed photon increases the electron density at this depth by one. The calculation starts with the intrinsic free carrier density of 10^16^ cm^−3^ and goes from −500 fs to 500 fs with respect to the laser peak. In contrast to linear absorption for the used flux of 10 mJ/cm^2^ during the laser pulse, about 82% of the laser energy is reflected and up to 30 times higher e-h pair density is generated. From the absorbed energy, 86% is deposited in a 10 nm surface layer of the sample. Such a narrow depth can much better explain the lattice behavior in the first 5 ps where expansion occurs in a similar depth close to the surface. Furthermore, the absorbed energy per volume close to the surface is increasing compared to linear absorption. Otherwise, such strong surface strain could also not be explained.

## IV. CONCLUSIONS

In conclusion, we found that the ultrashort excitation of semiconductors at high laser fluxes can only be well reproduced by considering time and depth dependent absorption of the pulse. Due to high e-h pair density, the excitation depth is more than one order of magnitude smaller than assuming linear absorption. The high e-h density gives rise to a lattice expansion of 4% at 2 ps after the excitation, which is close to the Lindemann limit of 10%.^24^ Fast recombination at the surface reduces the strain to one tenth of the initial strain after 10 ps. The results offer the possibility to observe transient properties like deformation potential and ambipolar carrier diffusion which are fundamental material properties used in our theoretical model on short time scales during nonequilibrium carrier transport in semiconductors.
